# Proprioceptive drift is affected by the intermanual distance rather than the distance from the body’s midline in the rubber hand illusion

**DOI:** 10.3758/s13414-020-02119-7

**Published:** 2020-09-10

**Authors:** Roberto Erro, Angela Marotta, Mirta Fiorio

**Affiliations:** 1 Department of Neurosciences, Biomedicine and Movement Sciences, Università di Verona, Via Casorati 43, 37131 Verona, Italy; 2 Department of Medicine, surgery and Dentistry “Scuola Medica Salernitana,” Neuroscience Section, University of Salerno, Baronissi, SA Italy

**Keywords:** Multisensory integration, Ownership, Peripersonal space, Rubber hand

## Abstract

In the rubber hand illusion (RHI), simultaneous brush stroking of a subject’s hidden hand and a visible rubber hand induces a transient illusion of the latter to “feel like it’s my hand” and a proprioceptive drift of the hidden own hand toward the rubber hand. Recent accounts of the RHI have suggested that the illusion would only occur if weighting of conflicting sensory information and their subsequent integration results in a *statistically plausible* compromise. In three different experiments, we investigated the role of distance between the two hands as well as their proximity to the body’s midline in influencing the occurrence of the illusion. Overall, the results suggest that the illusion is abolished when placing the two hands apart, therefore increasing the mismatch between the visual and proprioceptive modality, whereas the proximity of the two hands to the body’s midline plays only a minor role on the subjective report of the illusion. This might be driven by the response properties of visuotactile bimodal cells encoding the peripersonal space around the hand.

**Electronic supplementary material:**

The online version of this article (10.3758/s13414-020-02119-7) contains supplementary material, which is available to authorized users.

In the rubber hand illusion (RHI), simultaneous brush stroking of a subject’s hidden hand and a visible rubber hand induces a transient illusion of the latter to “feel like it’s my hand” (Botvinick & Cohen, 1998). The RHI can be implicitly measured as the localization bias (the so-called proprioceptive drift) of the perceived position of the subject’s own hand towards the rubber hand (Kilteni, Maselli, Kording, & Slater, 2015; Tsakiris & Haggard, 2005) and has been used to investigate and manipulate the sense of body ownership.

Recent accounts of the RHI postulate a three-way weighted integration between vision, touch, and proprioception and suggest that a number of “intermodal matching” are required for the illusion to occur, including the synchronicity of visual and tactile stimulation and the congruency between the anatomical position of the subject’s own hand and of the rubber hand (Costantini & Haggard, 2007; Tsakiris & Haggard, 2007). In fact, both the asynchronous visuotactile stimulation and the incongruent position of the rubber hand with respect to the subject’s hidden hand, abolish the illusion (Costantini & Haggard, 2007; Ehrsson, Spence, & Passingham, 2004; Ide, 2013; Tsakiris & Haggard, 2005). Another factor that has been recently construed to be fundamental for a successful illusion is the spatial proximity between the two hands. When the subject’s own hand and the rubber hand are far apart, the proprioceptive load felt at the shoulder and at the elbow would not match the expected load given by the position of the rubber hand, thus creating a visuoproprioceptive mismatch and, in turn, decreasing the illusion (Lloyd, 2007). It has also been suggested that the illusion would reduce because of the rubber hand being outside the peripersonal space around the subject’s hidden hand (Preston, 2013). However, previous research has found mixed results in this regard (Kalckert, Perera, Ganesan, & Tan, 2019). Some authors have demonstrated that by increasing the distance between the two hands more than 30 cm, the illusion is not typically experienced (Kalckert & Ehrsson, 2014b; Lloyd, 2007), whereas others found the RHI to be robust against distance manipulation (Abdulkarim & Ehrsson, 2016; Motyka & Litwin, 2019; Zopf, Savage, & Williams, 2010). Many factors that can account for such discrepancy, including the differences in the experimental setup. For example, Lloyd (2007) reported that distance between the two hands larger than 27.5 cm significantly reduces the illusion. However, in this experiment (Lloyd, 2007), the anatomical congruency between the two hands was not kept constant (i.e., the subject’s hidden hand was increasingly placed away from the rubber hand while being progressively rotated), and this, on its own, might have led to a decline in the experience of the illusion (Tsakiris & Haggard, 2005). Moreover, Preston (2013) demonstrated that the illusion is reduced only when the rubber hand is placed away from the subject’s hidden hand as well as from the trunk (i.e., the illusion would not decrease when the rubber hand is close to the trunk, despite being far from the subject’s hand), suggesting that the distance between the two hand is not the only spatial constraint for the RHI.

Therefore, to address this issue in more detail, we performed three experiments in which we manipulated both the distance between the two hands as well as the distance of each hand from the body’s midline, while keeping constant the anatomical position of the two hands. Thus, in the first experiment we used the classical setup of the RHI (Botvinick & Cohen, 1998), with the rubber hand placed medially to the subject’s hidden hand, and progressively shifted away the latter up to a distance of 48 cm from the body’s midline. In the second experiment, the position of the two hands was interchanged, thus keeping the subject’s hidden hand always close to the body’s midline and moving the rubber hand laterally. In the third experiment, both hands were shifted away from the body’s midline, while keeping their intermanual distance unchanged. In all experiments, beyond measuring the effects of spatial manipulation onto the implicit marker of the illusion (i.e., the proprioceptive drift), we also explored its subjective correlates, by administering the classic questionnaire of the RHI (Botvinick & Cohen, 1998).

## Materials and methods

### Participants

We performed three different experiments. For each experiment, 15 right-handed healthy participants were recruited. There were no differences in age (Experiment 1: 23.47 ± 3.09 years; Experiment 2: 23.93 ± 3.57 years; Experiment 3: 25.47 ± 3.70 years), *F*(2, 42) = 1.37, *p* = .27, and gender distribution (Experiment 1: eight females; Experiment 2: seven females; Experiment 3: six females; χ^2^ = 0.524, *p* = .770).

We computed the sample size in each experiment using G*Power 3.1 (Faul, Erdfelder, Lang, & Buchner, 2007), assuming an anticipated effect size *f* of 0.25 (which is considered as medium according to Cohen, 1988), α error probability of 0.05, power (1 − β error probability) of 0.95, correlation among repeated measures of 0.8, and nonsphericity correction ε of 1. For our experimental design (with six measurements; see below for more details), the resulting sample size was 12 in each experiment. We recruited more participants (*n* = 15) to prevent reduction in statistical power due to potential dropouts. This number was in line with previous studies using the same RHI outcomes (Tsakiris, 2010; Tsakiris & Haggard, 2005).

All participants gave written informed consent in accordance with the Declaration of Helsinki. The protocol was approved by the ethical committee of University of Verona.

### Experimental setup

**Fig. 1:**
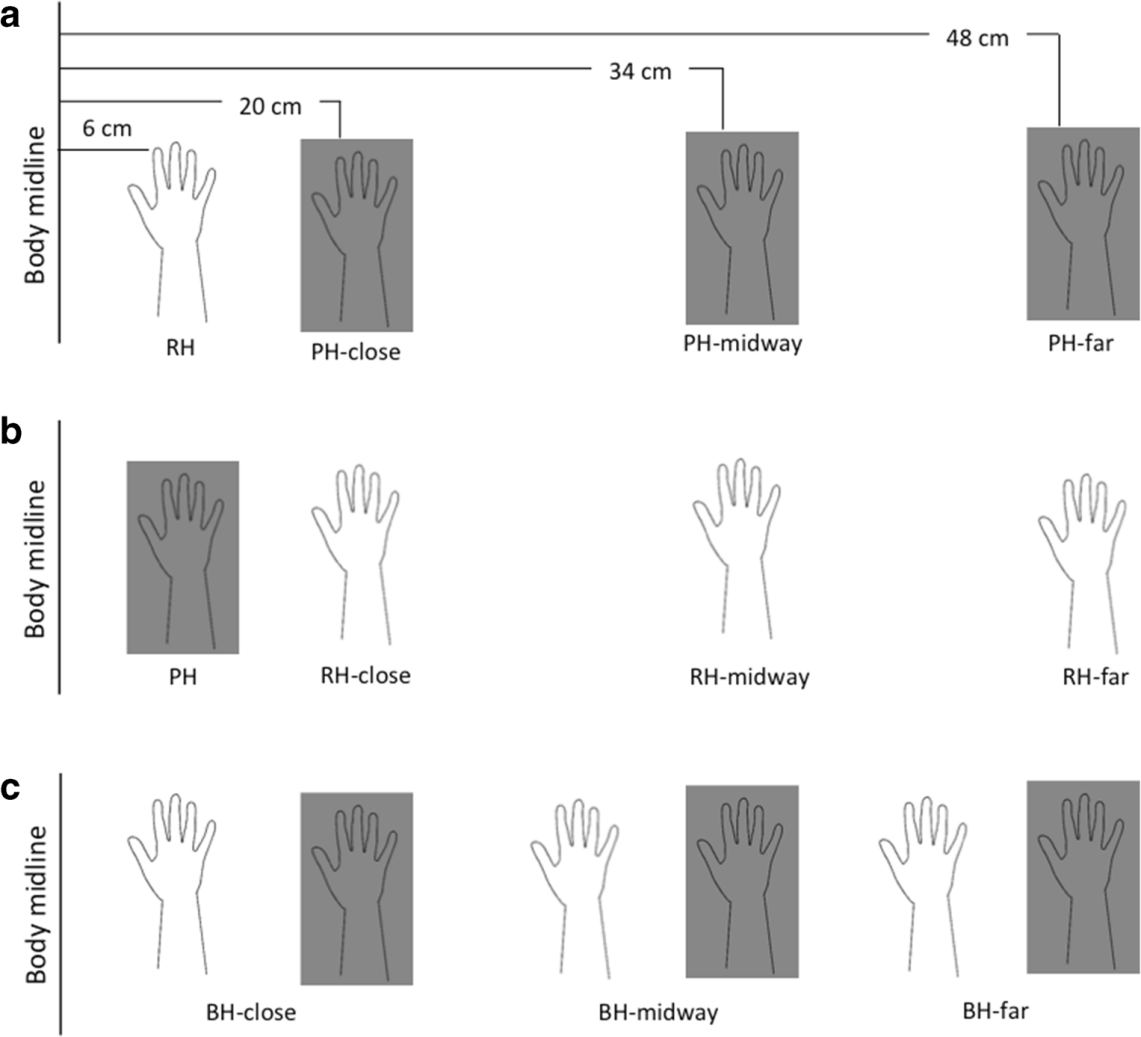
Experimental setup of the three experiments. In Experiment 1 (**a**), the rubber hand (RH) was placed 6 cm from the body’s midline, while the participant’s own hand (PH) was moved at three different distances laterally to the rubber hand: 20 (PH-close), 34 (PH-midway), and 48 cm (PH-far) from the midline. In Experiment 2 (**b**), the position of the hands was interchanged, and the RH was moved laterally to the participants’ hidden hand across the three conditions (RH-close, RH-midway, and RH-far). In Experiment 3 (**c**), both hands (BH) were moved laterally to the body’s midline. More precisely, based on the condition, the participant’s hand was placed close, midway, or far from the body’s midline, and the rubber hand was moved accordingly (BH close, BH midway, and BH far), by keeping constant the intermanual distance at 14 cm

The experimental setup was similar among the three experiments. Participants sat at a table with the right hand, palm facing down, concealed behind a black board. The left hand was kept on the left thigh. The entire upper body was covered by a cloth, to exclude any visual feedback from the arm and the trunk. The three experiments differed in terms of distance of the rubber and the participants’ hand from the body’s midline (see Fig. 1). More precisely, in Experiment 1, a realistic rubber hand was placed 6 cm from the body’s midline, while the participant’s own hand (PH) was moved three distances laterally to the rubber hand in three separate conditions: 20 cm (PH-close), 34 cm (PH-midway), and 48 cm (PH-far) from the body’s midline. In Experiment 2, the position of the hands was interchanged, and the rubber hand (RH) was moved laterally to the participants’ hidden hand across the three conditions (RH-close, RH-midway, and RH-far). In Experiment 3, both hands (BH) were moved laterally to the body’s midline. More precisely, based on the condition, the participant’s hand was placed close, midway, or far from the body’s midline, and the rubber hand was moved accordingly (BH-close, BH-midway, and BH-far), by keeping constant the intermanual distance (always 14 cm).

### Procedure

Each experiment lasted about 1 hour. Participants were asked to look at the rubber hand and to pay attention to any sensations while two small paintbrushes were used to synchronously or asynchronously stroke the rubber hand and the participants’ hand for 120 seconds. The stroking was applied on the tip of the index finger with a frequency of approximately 1 Hz. A 2 × 3 design was used with two stroking modalities (synchronous, asynchronous) and three conditions (close, midway, far). To avoid fatigue and learning effects, each stroking modality was applied only once in each condition.

### Measures of the illusion

As an implicit measure of the illusion, we used the proprioceptive drift—that is, a displacement in the perceived position of the participants’ hand toward to rubber hand. To obtain this measure, before and after each stroking modality, participants were asked to verbally report the location of their own index finger on a ruler placed in front of them, above a black board covering both the rubber hand and the participant’s hand (Fiorio et al., 2011). To avoid response bias, the starting position of the rule was changed trial by trial. The proprioceptive drift was computed as difference between the perceived position of the index finger before and after stimulation. A higher proprioceptive drift is generally observed after synchronous, but not asynchronous stroking (Botvinick & Cohen, 1998).

We also collected a subjective measure of the illusion by means of a nine-statement questionnaire (Botvinick & Cohen, 1998). After each stroking modality, participants were asked to indicate their agreement with each statement on a numerical rating scale (NRS) from −3 (*I totally disagree*) to +3 (*I totally agree*). Of note, the first three statements (embodiment-related statements: S1, S2, S3) refer to different aspects involved in the embodiment of the rubber hand. More precisely, S1 (“It seemed as if I felt the paintbrushes touching my finger where I saw the rubber hand being touched”) refers to an illusory localization of the felt touch over the rubber hand; S2 (“It seemed like the touch I felt was caused by the paintbrushes touching the rubber hand”) refers to a causal link between the vision and touch; S3 (“I felt as if the rubber hand was my own hand”) refers to the illusory feeling of ownership over the rubber hand. These statements usually receive higher scores after synchronous than after asynchronous stroking. The other six statements (control statements: S4 to S9) refer to perceptual effects that are less or unrelated to the embodiment of the rubber hand. They are used to control for participants’ compliance with the task and usually receive lower agreement scores compared with the embodiment-related statements. All statements are listed in Table S1 in the Supplementary Materials.

### Data analysis

**Fig. 2:**
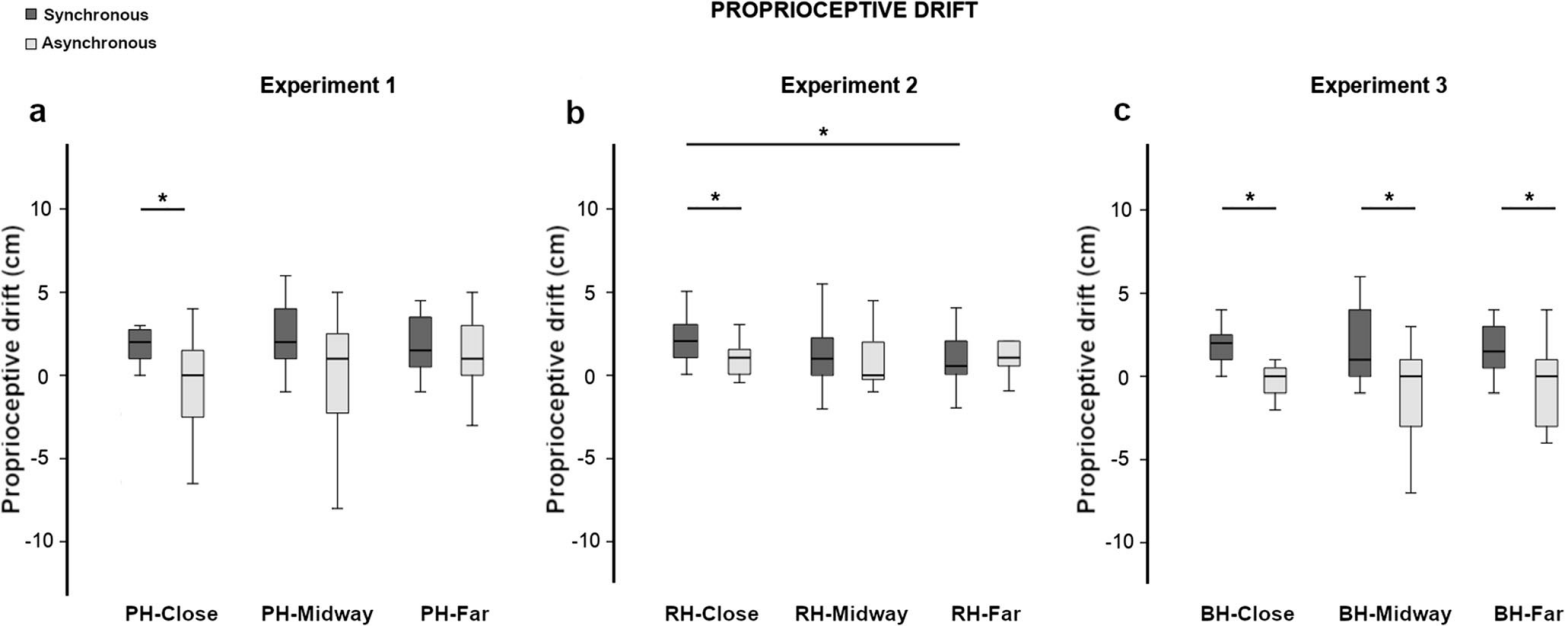
Boxplots of proprioceptive drift (in centimeters) in the three experiments. Proprioceptive drift was computed by subtracting proprioceptive judgements before and after stroking. The typical pattern of the RHI in the synchronous (dark-grey columns) compared with the asynchronous stroking (light-grey columns) was observed for the proprioceptive drift only in the close condition in Experiments 1 (**a**) and 2 (**b**). For Experiment 3 (**c**), instead, higher scores in the synchronous compared with the asynchronous stroking were observed in all the conditions (BH-close, BH-midway, BH-far). Of note, in Experiment 3, the intermanual distance was kept constant (i.e., 14 cm) across conditions. *Significant values (*p* < .05). Bars represent 1.5 times the interquartile range

**Fig. 3:**
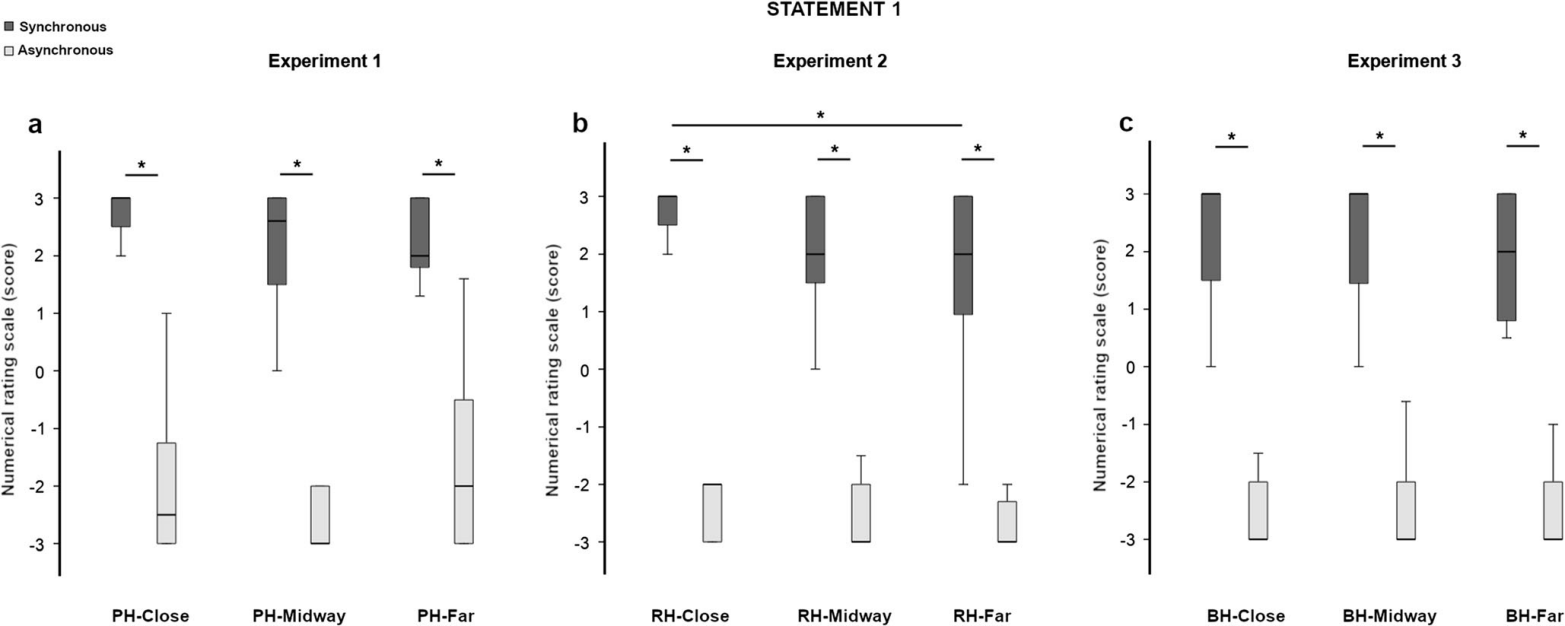
Boxplots of the scores at Statement 1 (“It seemed as if I felt the paintbrushes touching my finger where I saw the rubber hand being touched”) in the three experiments. The typical pattern of the RHI in the synchronous (dark-grey columns) compared with the asynchronous stroking (light-grey columns) was observed in all conditions and experiments. The position of the rubber hand significantly affected the score only in Experiment 2 (**b**), where participants gave higher scores at Statement 1 in the RH-close compared with the RH-far condition after synchronous stroking. *Significant values (*p* < .05). Bars represent 1.5 times the interquartile range

**Fig. 4:**
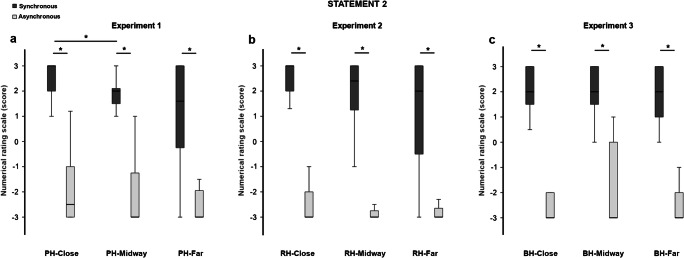
Boxplots of the scores at Statement 2 (“It seemed as though the touch I felt was caused by the paintbrushes touching the rubber hand”) in the three experiments. The typical pattern of the RHI in the synchronous (dark-grey columns) compared with the asynchronous stroking (light-grey columns) was observed in all conditions (close, midway, far) and experiments. The position of the rubber hand affected the scores in Experiment 1 (**a**), where participants gave higher scores at Statement 2 in the PH-close compared with the PH-midway condition after synchronous stroking. *Significant values (*p* < .05)

**Fig. 5:**
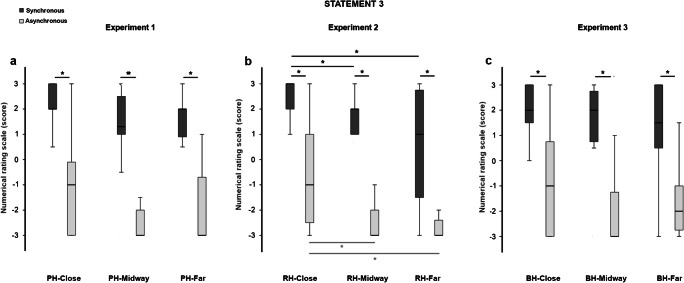
Boxplots of the scores at Statement 3 (“I felt as if the rubber hand was my own hand”) in the three experiments. The typical pattern of the RHI in the synchronous (dark-grey columns) compared with asynchronous stroking (light-grey columns) was observed in all the conditions (close, midway, far) and experiments. The position of the rubber hand affected the scores in Experiment 2 (**b**): participants gave higher scores at Statement 3 in the RH-close compared with the RH-midway and RH-far conditions after synchronous and asynchronous stroking. *Significant values (*p* < .05)

Statistical analyses were performed using SPSS^®^ Statistics (Version 19.0) and JASP (Version 0.12.2). Data were first checked for normality by means of the Shapiro–Wilk test. Since they were not normally distributed (Shapiro–Wilk test, *p* < .05) statistical analysis were performed using nonparametric tests. Data are represented as boxplots (see Figs. 2, 3, 4, and 5). Outlier values were included in the analysis. Median values, interquartile range and 95% confidence interval of all variables of interest are shown in Tables S2, S3, and S4 in the Supplementary Materials.

Questionnaire data and proprioceptive drift were analyzed stepwise. First, to verify the extent of illusion, we compared synchronous and asynchronous stroking separately for each condition by means of Wilcoxon signed-rank tests. Second, to explore potential differences between conditions, we applied the Friedman test separately for each stroking modality. A Wilcoxon signed-rank test was used for post hoc comparisons. To evaluate differences among the three experiments, we applied the Kruskal–Wallis test. Mann–Whitney *U* test was used for post hoc comparisons. Bonferroni correction was applied where necessary. Nonsignificant results for our main outcome measures (i.e., proprioceptive drift and embodiment-related statements) were further explored by means of Bayesian paired or independent-sample *t* tests. More precisely, Bayesian approach was used to better identify the conditions in which the results supported the null hypothesis. The results were expressed in terms of Bayes factors (BF_10_), where BF_10_ ≤ 0.33 indicates equal data distributions (i.e., the null hypothesis is supported), BF_10_ within 3 and 0.33 is considered as inconclusive (i.e., the Bayesian approach does not allow to confirm the null or the alternative hypothesis), and BF_10_ ≥ 3 indicates the different data distributions (i.e., the alternative hypothesis is supported). Finally, BF_10_ = 1 indicates that the null and the alternative hypothesis are equal (Lee & Wagenmakers, 2013; Scandola et al., 2019).

Moreover, we defined an illusion index for the proprioceptive drift, computed as the difference between the drift obtained in the synchronous and the asynchronous stroking (synchronous − asynchronous) separately for each experiment and condition. Similarly, the illusion index for the subjective measure of the illusion was computed as the difference between the score obtained in the synchronous and the asynchronous stroking (synchronous − asynchronous) for each embodiment-related statement. Description of the analyses and results for the illusion indexes are reported in the Supplementary Materials.

Within each experiment, a Spearman coefficient of correlation was used to assess any relation between proprioceptive drift and the embodiment-related statements (i.e., S1, S2m and S3) in all the conditions. The level of significance was set at *p* < .050. Effect size was estimated with Pearson’s *r* correlation (Cohen, 1988).

## Results

### Experiment 1

#### Proprioceptive drift

The proprioceptive drift was higher in the synchronous compared with the asynchronous stroking only in the PH-close condition (*Z* = −2.030, *p* = .042, *r* = .524; see Fig. 2a). No significant differences were found in the PH-midway (*Z* = −1.793, *p* = .073, *r* = .462) and PH-far (*Z* = −1.687, *p* = .092, *r* = .435) conditions, suggesting that the stroking modality did not affect proprioceptive drift in these conditions. To further test this hypothesis, we ran additional Bayesian comparisons between synchronous and asynchronous stocking separately for the PH-midway and PH-far conditions. The results were inconclusive in both cases (PH-midway BF_10_ = 2.086; PH-far BF_10_ = 1.063).

The Friedman test did not yield significant results in either the synchronous (χ^2^ = 1.345, *p* = .510) or in the asynchronous stroking (χ^2^ = 2.821, *p* = .244), indicating that the intermanual distance did not affect the amount of proprioceptive drift in the two stroking modalities. Regarding the synchronous stroking, Bayesian comparisons confirmed that data distributions were equal across conditions (PH-close vs. PH-midway BF_10_ = 0.311; PH-close vs. PH-far BF_10_ = 0.324; PH-midway vs. PH-far BF_10_ = 0.263). Similar results were found for comparison between PH-close and PH-midway conditions in the asynchronous stroking (BF_10_ = 0.292). Bayesian analyses yielded inconclusive results for the other comparisons (asynchronous: PH-close vs. PH-far BF_10_ = 1.818; PH-midway vs. PH-far BF_10_ = 0.530).

#### Questionnaire

##### Embodiment-related statements

The scores at the embodiment-related statements were higher after synchronous than after asynchronous stroking in all the tested conditions (S1: PH-close *Z* = −3.317, *p* = .001, *r* = .856; PH-midway *Z* = −3.429, *p* = .001, *r* = .885; PH-far *Z* = −3.412, *p* = .001, *r* = .881. S2: PH-close *Z* = −3.314, *p* = .001, *r* = .856; PH-midway *Z* = −3.200, *p* = .001, *r* = .826; PH-far *Z* = −2.556, *p* = .011, *r* = .660. S3: PH-close *Z* = −2.968, *p* = .001, *r* = .766; PH-midway *Z* = −3.213, *p* = .001, *r* = .830; PH-far *Z* = −3.115, *p* = .002, *r* = .804; see Figs. 3a, 4a, and 5a).

A significant effect of position was observed for S1 and S2, only in the synchronous stroking modality (S1, χ^2^ = 6.686, *p* = .035; S2, χ^2^ = 7.435, *p* = .024). Post hoc analysis (Bonferroni corrected critical *p* < .016) confirmed these results only for S2, which obtained significantly higher scores in the PH-close compared with the PH-midway condition (*Z* = −2.470, *p* = .014, *r =* .638; Fig. 4a). No other significant effect has been found (all comparisons, *p* > .025).

An additional Bayesian paired-samples *t* test revealed inconsistent results for S1 in the synchronous stroking (PH-close vs. PH-midway BF_10_ = 1.009; PH-close vs. PH-far BF_10_ = 1.103; PH-midway vs. PH-far BF_10_ = 0.352). Regarding the asynchronous stroking, the Bayes factor of BF_10_ = 0.279 clearly confirmed that scores at S1 were similar between the PH-close and PH-far conditions. The other comparisons, instead, yielded inconclusive results (asynchronous: PH-close vs. PH-midway BF_10_ = 0.420; PH-midway vs. PH-far BF_10_ = 0.977). For S2, Bayesian comparisons yielded inconclusive results in the synchronous stroking (PH-close vs. PH-far BF_10_ = 2.521; PH-midway vs. PH-far BF_10_ = 0.354). Regarding the asynchronous stroking, Bayesian analysis confirmed the null results for the comparisons between close and far conditions (PH-close vs. PH-far BF_10_ = 0.275) and between midway and far conditions (PH-midway vs. PH-far BF_10_ = 0.309). The Bayesian comparison between close and midway conditions was inconclusive (PH-close vs. PH-midway BF_10_ = 0.413).

Regarding S3, the null hypothesis was confirmed for the comparison between the midway and far conditions in the synchronous stroking (BF_10_ = 0.267). All other Bayesian comparisons yielded inconclusive results (synchronous: PH-close vs. PH-midway BF_10_ = 1.003; PH-close vs. PH-far BF_10_ = 1.328; asynchronous: PH-close vs. PH-midway BF_10_ = 0.843; PH-close vs. PH-far BF_10_ = 0.486; PH-midway vs. PH-far BF_10_ = 0.336).

##### Control statements

Regarding the control statements, we found higher scores in the synchronous compared with the asynchronous stroking for S4 in the PH-far condition (*Z* = −2.705, *p* = .007, *r* = .698), and S9 in the PH-close (*Z* = −2.549, *p* = .011, *r* = .658) and PH-midway conditions (*Z* = −2.552, *p* = .011, *r* = .659). Friedman tests did not yield significant results (all *p*s > .156).

### Experiment 2

#### Proprioceptive drift

Also, in Experiment 2, the proprioceptive drift was higher in the synchronous compared with the asynchronous stroking only in the RH-close condition (*Z* = −2.178, *p* = .029, *r* = .562; see Fig. 2b). No significant difference was found between synchronous and asynchronous stroking in the RH-midway (*Z* = −1.590, *p* = .112, *r* = .410) and RH-far (*Z* = −0.436, *p* = .663, *r* = .113) conditions, thus suggesting that proprioceptive drift was not affected by the stroking modality in these conditions. Additional Bayesian comparisons between synchronous and asynchronous stocking in the midway and far conditions showed inconclusive results (RH-midway BF_10_ = 2.086; RH-far BF_10_ = 1.063).

The Friedman test was significant in the synchronous stroking (χ^2^ = 7.191, *p* = .027). Post hoc comparisons (Bonferroni corrected critical *p* ≤ .016) showed that this was because of the higher drift in the RH-close compared with the RH-far condition (*Z* = −2.695, *p* = .007, *r* = .696; see Fig. 2b). The proprioceptive drift was not statistically different between the RH-close and the RH-midway conditions (*Z* = −1.147, *p* = .251, *r* = .296), and in the RH-midway and RH-far conditions (*Z* = −1.461, *p* = .144, *r* = .378). Bayesian paired-sample *t* tests revealed inconsistent results for these comparisons (RH-close vs. RH-midway BF_10_ = 0.380; RH-midway vs. RH-far BF_10_ = 0.643).

Friedman tests did not yield statistical significance in the asynchronous stroking (χ^2^ = 0.894, *p* = .640). Wilcoxon pairwise comparisons were not significant (all *p*s > .651). Bayesian factors confirmed that proprioceptive drift induced by asynchronous stroking was not statistically different across positions (RH-close vs. RH-midway BF_10_ = 0.271; RH-close vs. RH-far BF_10_ = 0.265; RH-midway vs. RH-far BF_10_ = 0.282).

#### Questionnaire

##### Embodiment-related statements

Similarly to Experiment 1, scores at the embodiment-related statements were higher in the synchronous compared with the asynchronous stroking modality in all conditions (S1: RH-close *Z* = −3.308, *p* = .001, *r* = .854; RH-midway *Z* = −3.360, *p* = .001, *r* = .868; RH-far *Z* = −3.192, *p* = .001, *r* = .824. S2: RH-close *Z* = −3.447, *p* = .001, *r* = .890; RH-midway *Z* = −3.142, *p* = .002, *r* = .811; RH-far *Z* = −3.210, *p* = .001, *r* = .829. S3: RH-close *Z* = −3.187, *p* = .001, *r* = .823; RH-midway *Z* = −3.213, *p* = .001, *r* = .830; RH-far *Z* = −3.066, *p* = .002, *r* = 0.792; see Figs. 3b, 4b, and 5b).

Friedman tests revealed a significant effect of condition for the synchronous stroking at all the embodiment-related statements (S1, χ^2^ = 8.914, *p* = .012; S2, χ^2^ = 6.864, *p* = .032; S3, χ^2^ = 9.347, *p* = .009). The significant result observed for S2 was not further confirmed at the post hoc analysis. For S1 and S3, instead, post hoc tests (Bonferroni-corrected critical *p* < .016) revealed higher scores in the RH-close compared with the RH-far condition (S1: *Z* = −2.429, *p* = .015, *r* = .627; S3: *Z* = −2.674, *p* = .007, *r* = .690; see Figs. 3b and 5b). Moreover, the score at S3 was higher in the RH-close compared with the RH-midway condition (*Z* = −2.896, *p* = .004, *r* = .748). A significant effect of condition was also observed in the asynchronous stroking for S1 (χ^2^ = 6.500, *p* = .039) and S3 (χ^2^ = 16.326, *p* = .001). Post hoc analysis confirmed this effect only for S3 with a higher score in the RH-close compared with the RH-midway (*Z* = −2.674, *p* = .008, *r* = .690) and the RH-far conditions (*Z* = −3.070, *p* = .002, *r* = .793; see Fig. 5b). Bayesian paired-samples *t* tests showed inconclusive results for all the embodiment-related statements in the synchronous stroking (S1: RH-close vs. RH-midway BF_10_ = 2.457; RH-midway vs. RH-far BF_10_ = 0.406; S2: RH-close vs. RH-midway BF_10_ = 1.660; RH-close vs. RH-far BF_10_ = 2.439; RH-midway vs. RH-far BF_10_ = 0.823; S3: RH-midway vs. RH-far BF_10_ = 0.462) and in the asynchronous stroking (S1: RH-close vs. RH-midway BF_10_ = 0.762; RH-close vs. RH-far BF_10_ = 1.367; RH-midway vs. RH-far BF_10_ = 0.526; S2: RH-close vs. RH-midway BF_10_ = 0.532; RH-close vs. RH-far BF_10_ = 0.270; RH-midway vs. RH-far BF_10_ = 0.504; S3: RH-midway vs. RH-far BF_10_ = 0.470).

##### Control statements

Higher scores were noted for some control statements after synchronous compared with the asynchronous stroking (S4: RH-close *Z* = −2.570, *p* = .010, *r* = .664. S7: RH-close *Z* = −2.866, *p* = .004, *r* = .740; RH-midway *Z* = −2.371, *p* = .018, *r* = .612. S9: RH-close *Z* = −2.521, *p* = .012, *r* = .651).

Friedman test yielded statistical significance in the synchronous stroking for S4 (χ^2^ = 9.116, *p* = .010), S7 (χ^2^ = 6.533, *p* = .038), and S9 (χ^2^ = 16.478*, p* < .001). As demonstrated by post hoc tests (Bonferroni-corrected critical *p* ≤ .016), this was because of the higher score in the RH-close compared with the RH-midway (S9: *Z* = −2.834*, p* = .005, *r* = .732) and the RH-far condition (S4: *Z* = −2.821, *p* = .005, *r* = .728; S7: *Z* = −2.595, *p* = .009, *r* = .670; S9: *Z* = −3.072, *p* = .002, *r* = .793). A significant effect of condition was observed in the asynchronous stroking for S9 (χ^2^ = 6.343, *p* = .042). This was because of higher scores in the RH-close compared with the RH-far conditions (*Z* = −2.499, *p* = .012, *r* = .645). Finally, we also found a significant effect of condition in both the synchronous (χ^2^ = 6.059, *p* = .048) and asynchronous stroking (χ^2^ = 7.103, *p* = .029) for S5. Post hoc analysis (Bonferroni-corrected critical *p* < .016), however, did not confirm these results (all *p*s > .018).

### Experiment 3

#### Proprioceptive drift

The proprioceptive drift was higher in the synchronous compared with the asynchronous stroking in all conditions (BH-close *Z* = −3.066, *p* = .002, *r* = .792; BH-midway *Z* = −2.391, *p* = .017, *r* = .617; BH-far *Z* = −2.485, *p* = .013, *r* = .642; see Fig. 2c).

Friedman test was not significant in both synchronous (χ^2^ = 0.143, *p* = .931) and asynchronous stroking (χ^2^ = 0.778, *p* = .678). To better characterize the lack of significant effect of distance of both hand from the body’s midline, we ran additional descriptive pairwise comparisons that confirmed the lack of significant effect of condition (synchronous: BH-close vs. BH-midway *Z* = −0.141, *p* = .888; BH-close vs. BH-far *Z* = −0.343, *p* = .731; BH-midway vs. BH-far *Z* = −0.140, *p* = .888; asynchronous: BH-close vs. BH-midway *Z* = −0.977, *p* = .329; BH-close vs. BH-far *Z* = 0.347, *p* = .729; BH-midway vs. BH-far *Z* = .542, *p* = .588). These findings suggest that the distance of the two hands from the body’s midline did not affect the amount of proprioceptive drift in the synchronous and asynchronous stroking. These findings were further confirmed by Bayesian paired-samples *t* tests. Bayesian factors, indeed, supported the null hypothesis (no differences between conditions) in both synchronous (BH-close vs. BH-midway BF_10_ = 0.263; BH-close vs. BH-far BF_10_ = 0.269; BH-midway vs. BH-far BF_10_ = 0.272) and asynchronous stroking (BH-close vs. BH-midway BF_10_ = 0.396; BH-close vs. BH-far BF_10_ = 0.282; BH-midway vs. BH-far BF_10_ = 0.283).

#### Questionnaire

##### Embodiment-related statements

The score at the embodiment-related statements was higher in the synchronous compared with the asynchronous stroking in all conditions (S1: BH-close *Z* = −3.455, *p* = .001, *r* = .892; BH-midway *Z* = −3.436, *p* = .001, *r* = .887; BH-far *Z* = −3.420, *p* = .001, *r* = .883; S2: BH-close *Z* = −3.425, *p* = .001, *r* = .884; BH-midway *Z* = −3.421, *p* = .001, *r* = .883; BH-far *Z* = −3.314, *p* = .001, *r* = .856; S3: BH-close *Z* = −3.068, *p* = .002, *r* = .792; BH-midway *Z* = −3.184, *p* = .001, *r* = .822; BH-far *Z* = −2.906, *p* = .004, *r* = .750; see Figs. 3c, 4c, and 5c). Friedman tests did not yield significant results in both synchronous (S1: χ^2^ = 4.789, *p* = 0.091; S2: χ^2^ = 5.688, *p* = .058; S3: χ^2^ = 4.054, *p* = .132) and asynchronous stroking (S1: χ^2^ = 0.923, *p* = .630; S2: χ^2^ = .074, *p* = .964; S3: χ^2^ = 2.743, *p* = .254). Descriptive post hoc comparisons (Bonferroni-corrected critical *p* < .016) confirmed these findings (all *p*s > .034). For S1, Bayesian paired-samples *t* test supported the null hypothesis for the comparisons between BH-close and BH-midway conditions in the synchronous stroking (BF_10_ = 0.268) and for all comparisons in the asynchronous stroking (BH-close vs. BH-midway BF_10_ = 0.263; BH-close vs. BH-far BF_10_ = 0.294; BH-midway vs. BH-far BF_10_ = 0.296). The other comparisons showed inconclusive results (synchronous: BH-close vs. BH-far BF_10_ = 1.465; BH-midway vs. BH-far BF_10_ = 1.263). With regards to S2, Bayesian factors supported the null hypothesis for comparison between BH-close and BH-midway conditions in the synchronous stroking (BF_10_ = 0.268) and between BH-close and BH-far conditions in the asynchronous stroking (BH-close vs. BH-far BF_10_ = 0.268). Other comparisons showed inconclusive Bayesian factors (synchronous: BH-close vs. BH-far BF_10_ = 1.153; BH-midway vs. BH-far BF_10_ = 2.876; asynchronous: BH-close vs. BH-midway BF_10_ = 0.396; BH-close vs. BH-far BF_10_ = 0.268; BH-midway vs. BH-far BF_10_ = 0.362). Finally, for S3, the Bayesian factors were inconclusive in most cases (synchronous: BH-close vs. BH-midway BF_10_ = 1.896; BH-close vs. BH-far BF_10_ = 0.378; asynchronous: BH-close vs. BH-midway BF_10_ = 1.227; BH-midway vs. BH-far BF_10_ = 0.700). Support to the null hypothesis was found for the comparisons between midway and far condition in the synchronous stroking (BH-midway vs. BH-far BF_10_ = 0.263) and between close and far conditions in the asynchronous stroking (BH-close vs. BH-far BF_10_ = 0.306).

##### Control statements

We found higher scores after synchronous than asynchronous stroking at S7 (BH-close *Z* = −2.264, *p* = .024, *r* = .585; BH-far *Z* = −2.023, *p* = .043, *r* = .522) and S9 (BH-close Z = −2.243, *p* = .025, *r* = .579; BH-midway *Z* = −2.264, *p* = .024, *r* = .585; BH-far *Z* = −2.214, *p* = .027, *r* = .572). Friedman tests were not significant (all *p*s > .292).

### Comparisons among the experiments

#### Proprioceptive drift

The proprioceptive drift was similar among the experiments in both synchronous (close χ^2^ = 0.134, *p* = .935; midway χ^2^ = 1.274, *p* = .529; far χ^2^ = 2.772, *p* = .250) and asynchronous stoking (close χ^2^ = 3.865, *p* = 0.145; midway χ^2^ = 2.329, *p* = .312; far χ^2^ = 4.814, *p* = .090). Descriptive independent sample comparisons (Bonferroni-corrected critical *p* < .016) confirmed these findings (all *p*s > .03). Bayesian independent-samples *t* tests showed inconclusive results (0.347 < BF_10_ < 2.192).

#### Embodiment-related statements

Kruskal–Wallis test revealed no differences among experiments for S1 (synchronous all conditions, *p* > .399; asynchronous all conditions, *p* > .284), S2 (synchronous all conditions, *p* > .384; asynchronous all conditions, *p* > .288), and S3 (synchronous all conditions, *p* > .369; asynchronous all conditions, *p* > .057). These nonsignificant results were also confirmed by Mann–Whitney *U* tests (all *p*s > .130). Bayesian independent-samples *t* tests showed inconclusive results (all 0.344 < BF_10_ < 2.880).

### Correlations

No significant correlations have been found between proprioceptive drift and scores at embodiment-related statements for both synchronous (Experiment 1: *p* > .407; Experiment 2: *p* > .060; Experiment 3: *p* > .190) and asynchronous stroking (Experiment 1: *p* > .279, Experiment 2: *p* > .100, Experiment 3: *p* > .170). These findings suggest that objective and subjective measures of illusion were independent from each other.

## Discussion

The main finding of the current work is that the expected proprioceptive recalibration of the subjects’ hidden hand after synchronous stroking, which is measured by means of the so-called proprioceptive drift, is strongly modulated by the distance between the two hands. In other words, placing the two hands far apart and therefore increasing the “mismatch” between the visual and proprioceptive modality, abolishes the illusion in terms of objective measures, as demonstrated in Experiments 1 and 2, when a significant proprioceptive drift occurred only with the two hands being close to each other. On the contrary, when the intermanual distance was kept constant (Experiment 3), a significant and similar proprioceptive recalibration occurred in all conditions, suggesting that the proximity of the two hands to the body’s midline only plays a minor role in terms of the implicit measure of the illusion.

Recent accounts of the RHI postulate that the perception of body ownership (and its experimental modulation) is governed by Bayesian sensory inference, thereby conflicting sensory information are integrated to minimize variance in the final sensory estimate (Ehrsson & Chancel, 2019; Fang, et al., 2019; Samad, Chung, & Shams, 2015). Accordingly, the illusion would decrease or vanish whenever weighting of conflicting sensory information and their subsequent integration does not result in a *statistically plausible* compromise (Erro, Marotta, Tinazzi, Frera, & Fiorio, 2018; Fuchs, Riemer, Diers, Flor, & Trojan, 2016) as in the case with asynchronous stimulation or with an incongruent position of the rubber hand with respect to the subject’s hidden hand. Here, we demonstrate that this holds true also when the two hands are increasingly placed apart, which arguably creates a mismatch between the visual and proprioceptive modalities. In fact, in both Experiments 1 and 2, the proprioceptive load felt at the shoulder and elbow would not arguably match the expected one, based on the visual modality (i.e., the position of the rubber hand). Our findings are in line with some (Kalckert et al., 2019; Lloyd, 2007; Preston, 2013), but not all (Abdulkarim & Ehrsson, 2016; Zopf et al., 2010) previous results. Overall, it is difficult to compare our results with those previously obtained because of the difference in the experimental setup used in each of these studies. On the one hand, our findings confirm and expand on those obtained by a body of previous research (Kalckert & Ehrsson, 2014b; Lloyd, 2007; Preston, 2013). Lloyd (2007) found that the illusion declines already at 27.5 cm distance between hands, which mirrors what we found when the hands were placed 28 cm apart (i.e., midway position; see Fig. 1), in both Experiments 1 and 2. However, the spatial arrangement of the hands in Lloyd’s (2007) study resulted not only in varying the lateral distance between the hands but also the anatomical congruency, being that the rubber hand progressively rotated with increasing distances between the hands. Moreover, for distances of more than 37.5 cm, the rubber hand crossed the subject’s body’s midline and was placed in the contralateral hemispace (compare Lloyd, (2007, Fig. 1). Both factors (anatomical congruency and location in the contralateral hemispace) could have contributed to reduce the illusion in the study by Lloyd (2007), whereas these confounding factors were not an issue in the current study, as the two hands were in the same anatomical position and placed in the same subject’s hemispace in all experiments. Our results are also largely in agreement with Kalckert and Ehrsson (2014b), who found a significant decrease of the illusion when the hands were placed 27 cm apart (which corresponds to our midway position; see Fig. 1) both in terms of implicit (i.e., proprioceptive drift) and explicit (i.e., ownership ratings) measures, albeit with some degree of dissociation between them, as observed in the current study. One difference is that we found the illusion to be present in terms of ownership ratings even at higher distances (i.e., far position), which was not observed in the study by Kalckert and Ehrsson (2014b). It should be noted, however, that other factors could have influenced this finding. In fact, in the current study we used a horizontal rather than a vertical setup (Kalckert & Ehrsson, 2014b), thus allowing us to manipulate not only the relative position between the hands but also the distance of the two hands from the body’s midline, a factor that cannot be modulated with a vertical setup. Preston (2013) found that both the distance between the two hands and the relative distance between the rubber hand and the subject’s body’s midline are important factors influencing the illusion. In fact, she found that the illusion reduced only when the rubber hand was shifted away from both the subject’s hand and the subject’s trunk, but not when the real hand was moved away, being that the rubber hand kept in a constant position near the subject’s midline (Preston, 2013). This is very similar to what has been observed here in Experiments 1 and 2. However, we also tested in Experiment 3 the possibility that the distance from the body’s midline as such, while keeping constant the intermanual distance, could directly influence the illusion and found no evidence for this. Therefore, our findings are in agreement with Preston (2013) arguing that the peripersonal space (PPS) is important for the subjective report of the RHI, but also expand on her results showing that the proximity of the two hands, which implies a hand-centered representation of the PPS, is a crucial factor for the illusion to occur. In sum, the current study is in line with previous evidence showing an effect of distance between the real hand and the rubber hand in the RHI (Kalckert & Ehrsson, 2014b; Lloyd, 2007; Preston, 2013).

On the other hand, other research argued against a role of the lateral distance between the two hands in modulating the illusion. However, in the study of Abdulkarim and Ehrsson (2016), an apparatus was devised to laterally displace the participant’s hidden hand during the synchronous stroking (either towards or away from the rubber hand) by a maximum of 8 cm from the initial position. It could well be that this little modulation of only 8 cm might have been insufficient to drive significant changes of proprioceptive drift. Larger distances between the two hands have been instead investigated in the study by Zopf et al. (2010), and no significant changes were observed in terms of position judgment of the subjects’ hidden hand across different conditions with short (15 cm) versus large (45 cm) lateral distance, although the lack of difference between the synchronous and asynchronous conditions at the closest distance makes it hard to interpret these findings. Moreover, it is interesting to note that the lateral distance influenced the “importance” of other modalities in the induction of the illusion (Zopf et al., 2010). That is, the closer the hands were, reflecting a low visuoproprioceptive mismatch, the higher incongruity in other modalities (i.e., tactile) was allowed, and vice versa. The further the hands were apart, the more the visuotactile synchronicity became important in the induction of the illusion. Therefore, their findings would suggest that the lateral distance plays a role in the multisensory integration process occurring during the RH, even though they failed to demonstrate a direct effect on the proprioceptive drift (Zopf et al., 2010). Notably, however, in the study of Zopf et al. (2010), the stroking phase lasted 2.5 minutes and was repeated three times before the poststroking proprioceptive judgement. This methodological choice would have strengthened the illusion, thus preventing the drop of proprioceptive drift despite the larger distance between the participant’s and the rubber hand.

Altogether, our and prior results would support the argument that there exist spatial constraints of the RHI—namely, the proximity between the two hands, which could reflect a hand-centered representation of the PPS. Beyond the limits of this representation, the illusion would tend to decline. Evidence for hand-centered coding of visual and proprioceptive stimuli surrounding the hand comes from a recent fMRI study using the RHI (Brozzoli, Gentile, & Ehrsson, 2012). This study linked the subjective feeling of ownership and the proprioceptive drift to perihand space remapping in the premotor cortex and posterior parietal cortex, respectively (Brozzoli et al., 2012). Interestingly, previous studies linked the activity of bimodal visuotactile neurons to these hand-centered representation of the PPS (Graziano & Gross, 1998; Graziano, Hu, & Gross, 1997). These neurons are spatially anchored to the limb, follow changes in limb position (Graziano & Gross, 1998; Graziano et al., 1997), and are involved in multisensory representation of the body and its surrounding (Guterstam, Zeberg, Özçiftci, & Ehrsson, 2016). Our results are consistent with the evidence that the activity of visuotactile bimodal neurons in the premotor and posterior parietal cortices, implicated in the multisensory encoding of limb position, persists even when the visual inputs is absent (i.e., after the hand has been hidden; Obayashi, Tanaka, & Iriki, 2000), and that their visual receptive fields are anchored to the hand and extend into the space adjacent to the skin surface (i.e., perihand space) up to 40 cm (Fogassi et al., 1996; Graziano & Gross, 1996; Graziano et al., 1997; Guterstam et al., 2016). Increasing the lateral distance beyond this limit would take the visual representation of the rubber hand outside the visuotactile receptive fields of the subjects’ hidden hand and therefore decrease the illusion.

Another interesting finding obtained in our study was the dissociation between the objective (i.e., proprioceptive drift) and subjective (i.e., questionnaire) correlates of the RHI, as also confirmed by the lack of significant correlations. These results were further explored by means of additional analyses in which we examined the correlations between the proprioceptive shift and the illusion indexes for the embodiment-related statements (see Supplementary Materials). We found a significant positive correlation between the proprioceptive shift and the illusion index of S3 in the midway condition of Experiment 1 (see Supplementary Materials), which would be in line with previous studies, showing that the proprioceptive drift correlates with the subjective measure of the illusion (e.g., Abdulkarim & Ehrsson, 2016; Kalckert & Ehrsson, 2014a). Nonetheless, in all other conditions and experiments, the correlations did not reach the statistical threshold, which largely mirrors previous results (Fiorio et al., 2011; Tsakiris & Haggard, 2005). The question of whether an association between the objective and subjective measures of the illusion exists therefore remains open and warrants future research.

We further demonstrated here that the subjective reports of the illusion are not grossly influenced by either the intermanual distance or the proximity to the body’s midline. However, as mentioned above, we found a significant decrease of the strength of the illusion in Experiment 2, when progressively placing the rubber hand away from both the body’s midline and from the subjects’ hidden hand, a finding that did not occur in Experiment 1. Since Experiments 1 and 2 are specular to each other, implying that in both there is the same degree of visuoproprioceptive mismatch for each condition (i.e., close, midway, far), additional factors should have accounted for this asymmetry in the two experiments. The drop in the ownership statements in Experiment 2 would be in line with previous results (Preston, 2013) and might suggest that the illusion strength has decreased because of the rubber hand approaching to the limits of both peritrunk and perihand spaces, a condition that did not happen in Experiment 1, where the rubber hand was placed in between the trunk and the subjects’ hidden hand (Fig. 1). In the latter case, despite the rubber hand being arguably placed outside the subject’s perihand space in the “far condition,” it was well located within the overall PPS and, most importantly, near to the subjects’ body. This sort of facilitation might reflect the evidence that the visual responses of neurons of the “PPS network” lie primarily within a head–face and/or arm–hand centered somatosensory representation of the body (Brozzoli, Ehrsson, & Farnè, 2014). The proximity of the rubber hand to the subject’s body in Experiment 1 would have facilitated the multisensory integration, with a head-face-trunk representation of the PPS prevailing onto a hand-centered one in driving the subjective report of ownership. Conversely, the results from Experiments 2 and 3 show that the subjective sense of ownership as well as the proprioceptive recalibration were primarily influenced by the proximity of the two hands, which implies a hand-centered representation of the PPS. Therefore, whereas our and previous results (Kalckert & Ehrsson, 2014b; Lloyd, 2007; Ehrsson et al., 2004; Tsakiris & Haggard, 2005) about the spatial constraints of the RHI imply and support the involvement of a hand-centered representation of the PPS, we suggest that there might be a dynamic modulation of the PPS representation, further supporting the argument that integration of signals from different sensory modalities might be facilitated within near-personal space (Makin, Holmes, & Ehrsson, 2008; Spence, Pavani, & Driver, 2004a; Spence, Pavani, Maravita, & Holmes, 2004b). This dynamic modulation of the PPS would adapt to the actual body position in space, and the embodiment of external objects, in turn, would depend on whether they are within or outside both the peritrunk and perihand space (i.e., in a *plausible* location with respects of both the trunk and the arm). This ties in nicely with the evidence in monkeys that premotor neurons generate multiple representations of space, which are centered on different body parts (i.e., head centered, arm centered, etc.), follow changes in limb position, and are modulated by both visual and proprioceptive information (Graziano, 1999; Graziano & Gross, 1993; Graziano et al., 1997).

In summary, our results argue for a spatial limit of the RHI illusion, whereby the proprioceptive drift diminishes or vanish as a function of increased distance between the two hands. This might reflect the response properties of visuotactile bimodal cells encoding the peripersonal space around the hand (Graziano & Gross, 1996; Graziano et al., 1997). On the other side, the subjective experience of embodiment is less influenced by this parameter and seems to relate to a more complex representation of the overall space around our body, resulting from the interaction of different body-part-centered PPS representations and forming a multisensory structure to guide actions directed to objects within reaching distance (di Pellegrino & Làdavas, 2015).

## Electronic supplementary material

ESM 1 (DOCX 53 kb)
