# Supplementary material for: Proprioceptive drift is affected by the intermanual distance rather than the distance from the body’s midline in the rubber hand illusion
Source: Atten Percept Psychophys. 2020 Sep 10;82(8):4084–95. doi: 10.3758/s13414-020-02119-7 (PMC7593391; doi:10.3758/s13414-020-02119-7)
Supplement: Supplementary file 1 — (DOCX 53 kb) [file 13414_2020_2119_MOESM1_ESM.docx]

Supplementary material

**Proprioceptive drift is affected by the intermanual distance rather than the distance from the body’s midline in the rubber hand illusion**

**Roberto Erro*, Angela Marotta*, Mirta Fiorio**

^*^These authors equally contributed to this work.

**Correspondence:** Mirta Fiorio ([mirta.fiorio@univr.it](mailto:mirta.fiorio@univr.it)) and Roberto Erro ([rerro@unisa.it](mailto:rerro@unisa.it))

**Data analysis on the illusion index**

To further characterize the potential effect of the distance between hands and from the body’s midline on the RHI, we computed and analyzed an index of illusion, defined as the difference between the synchronous and asynchronous stroking. This index represents the amount of proprioceptive drift or subjective experience of ownership in the synchronous condition with respect to the asynchronous condition (`Abdulkarim & Ehrsson, 2016; Grynberg & Pollatos, 2015; Tsakiris et al., 2011). The illusion index for the proprioceptive drift (called proprioceptive shift) was computed by subtracting the proprioceptive drift in the asynchronous from that in the synchronous stroking (synchronous – asynchronous; `Abdulkarim & Ehrsson, 2016). Similarly, the illusion index for the subjective measure of the illusion was calculated for each embodiment-related statement (i.e., S1, S2, S3) by subtracting the scores in the asynchronous from the scores in the synchronous stroking (synchronous − asynchronous), thus obtaining three indexes (ΔS1, ΔS2, ΔS3). Higher scores in these measures are suggestive of a stronger illusion when the visual and tactile simulations are synchronous (Grynberg & Pollatos, 2015). The illusion index for the proprioceptive drift and for the subjective measure was computed separately in each condition (close, midway, and far) in each experiment (1, 2, and 3).

Friedman test was applied to compare the illusion index across conditions (close, midway, and far), separately for each experiment. A Wilcoxon signed-rank test was used for post hoc comparisons. Moreover, the illusion index obtained in each condition was compared across the three experiments by means of a Kruskal–Wallis test. The Spearman coefficient of correlation was used to explore the relation between proprioceptive shift and the illusion index for the embodiment-related statements (ΔS1, ΔS2, ΔS3) in all the conditions. Post hoc comparisons were conducted by means of a Mann–Whitney *U* test. Bonferroni correction was applied when necessary.

**Results from the illusion index**

**Illusion index for proprioceptive drift.** Friedman test did not yield significant results in any of the experiments (Experiment 1: χ^2^ = 2.140, *p* = .343; Experiment 2: χ^2^ = 4.769, *p* = .092; Experiment 3: χ^2^ = 1.0521, *p* = .591). To better characterize these findings, we ran additional post hoc analyses (Bonferroni-corrected critical *p* < .016). In all the experiments, pairwise comparisons further confirmed that the proprioceptive shift did not significantly vary across conditions (all comparisons, *p* > .049). Bayesian analyses provided support in favor of the null hypothesis for comparisons between close and midway conditions of Experiments 1 (BF_10_ = 0.263) and 2 (BF_10_ = 0.310) and for comparisons between all conditions of Experiment 3 (close vs. midway: BF_10_ = 0.325; close vs. far: BF_10_ = 0.265; midway vs. far: BF_10_ = 0.290). For all other cases, Bayesian analyses revealed inconclusive results (0.310 < BF_10_ < 1.820).

Interestingly, by comparing the proprioceptive shift between the three experiments, Kruskal–Wallis tests yielded a significant result only in the far condition (χ^2^ = 7.654, *p* = .022). This was due to a higher proprioceptive shift in Experiment 3 compared with Experiment 2 (*Z* = −2.540, *p* = .011), suggesting that the intermanual distance is a crucial factor for the proprioceptive component of the illusion. No other significant difference was found (all *p*s > .063). In all cases in which the frequentist approach showed nonsignificant results between experiments, Bayesian analyses showed inconclusive results (all 0.743 < BF_10_ < 1.083).

Overall, these findings provide further evidence on the minor role of the distance from the body’s midline in influencing the proprioceptive recalibration of the participant’s hand towards the rubber hand.

**Illusion index for the subjective measure.** Friedman test did not yield significant results in any of the experiments for ΔS1 (all comparisons, *p* > .258), ΔS2 (all comparisons, *p* > .080), and ΔS3 (all comparisons, *p* > .557), thus suggesting that the amount of subjective illusion induced by synchronous compared with asynchronous stroking was not affected by the condition. Descriptive pairwise comparisons (Bonferroni-corrected critical *p* < .016) confirmed these findings for Experiment 1 (all comparisons, *p* > .091), Experiment 2 (all *p*s > .021), and Experiment 3 (all comparisons, *p* > .192).

Regarding ΔS1, Bayesian factor analyses provided support for the null hypothesis for the comparison between close and midway conditions of Experiment 1 (BF_10_ = 0.265), Experiment 2 (BF_10_ = 0.328), and Experiment 3 (BF_10_ = 0.265). For ΔS2, support for the null hypothesis was found for the comparison between close and midway conditions of Experiment 1 (BF_10_ = 0.332), while the Bayes factor of BF_10_ = 3.421 supported the alternative hypothesis for comparison between close and far conditions of Experiment 2. Finally, for ΔS3, Bayesian analyses provided support for the null hypothesis for comparisons between close and midway conditions in Experiment 1 (BF_10_ = 0.280), and in Experiment 2 (BF_10_ = 0.273), between close and far conditions in all experiments (Experiment 1: BF_10_ = 0.274; Experiment 2: BF_10_ = 0.275; Experiment 3: BF_10_ = 0.267) and between midway and far conditions in Experiment 1 (BF_10_ = 0.274) and Experiment 2 (BF_10_ = 0.319). In all other cases, Bayesian factors were inconclusive (0.359 < BF_10_ < 1.865).

Kruskal–Wallis tests did not reveal significant differences across the three experiments for any of the subjective indexes (all *p*s > .353). These findings were further corroborated by post hoc Mann–Whitney *U* tests, which showed not significant results (all *p*s > .182). In all the cases, Bayesian independent-samples *t* tests were inconclusive (all 0.345 < BF_10_ < 0.805).

The analyses of the illusion index further support the lack of significant effect of distance in Experiment 3.

**Correlations.** A significant positive correlation between the proprioceptive shift and the ΔS3 was found in the midway condition of Experiment 1 (*p* = .012. *r* = .627). No other significant correlations have been found (Experiment 1: *p* > .153; Experiment 2: *p* > .521; Experiment 3: *p* > .105). These results further corroborate the lack of correlations between objective and subjective measures of the rubber hand illusion observed in the main analyses.

**Table S1** Embodiment-related and control statements

| **Illusion-related statements** |
| --- |
| S1. It seemed as if I were feeling the touch of the paintbrushes in the location where I saw the rubber hand touched |
| S2. It seemed as though the touch I felt was caused by the paintbrushes touching the rubber hand |
| S3. I felt as if the rubber hand was my own hand |
| **Control statements** |
| S4. It felt as if my hand were drifting toward the rubber hand |
| S5. It seemed as if I might have more than one hand or arm |
| S6. It seemed as if the touch I was feeling came from somewhere between my own hand and the rubber hand |
| S7. It felt as if my hand were turning rubbery |
| S8. It appeared as if the rubber hand were drifting towards my hand |
| S9. The rubber hand began to resemble my own hand |

| **Table S2.** Median (interquartile range [IQR]) and 95% confidence interval (95% CI) of proprioceptive drift, embodiment-related statements, and control statements in Experiment 1 | | | | | | |
| --- | --- | --- | --- | --- | --- | --- |
|  | **Conditions** | | | | | |
|  | PH-close | | PH-midway | | PH-far | |
| **Drift** | Median (IQR) | 95% CI | Median (IQR) | 95% CI | Median (IQR) | 95% CI |
| Synchronous | 2.00 (2.00) | 0.69, 3.23 | 2.00 (4.00) | 0.97, 4.22 | 1.50 (4.00) | 0.62, 4.44 |
| Asynchronous | 0.00 (5.00) | −2.36,1.16 | 1.00 (5.50) | −2.16, 2.02 | 1.00 (3.00) | −0.08, 2.61 |
|  | | | | | | |
| **Embodiment-related statements** | | | | | | |
| **Statement 1** |  | |  | |  | |
| Synchronous | 3.00 (1.00) | 2.32, 3.00 | 2.60 (2.00) | 1.49, 2.72 | 2.00 (1.40) | 1.12, 2.70 |
| Asynchronous | −2.50 (2.00) | −2.76, −0.82 | −3.00 (1.00) | −2.97, −1.58 | −2.00 (3.00) | −2.53, −0.74 |
|  |  |  |  |  |  |  |
| **Statement 2** |  |  |  |  |  |  |
| Synchronous | 3.00 (4.00) | 1.70, 2.92 | 2.00 (1.20) | 0.45, 2.35 | 1.60 (3.50) | −0.13, 2.33 |
| Asynchronous | −2.50 (2.00) | −2.64, −0.61 | −3.00 (2.00) | −2.75, −1.39 | −3.00 (1.50) | −3.00, −0.58 |
|  |  |  |  |  |  |  |
| **Statement 3** |  |  |  |  |  |  |
| Synchronous | 2.00 (2.00) | 1.58, 2.66 | 1.30 (2.00) | 0.42, 2.22 | 2.00 (1.20) | 0.19, 2.25 |
| Asynchronous | −1.00 (3.80) | −2.25, 0.21 | −3.00 (1.00) | −2.84, −1.43 | −3.00 (2.60) | −2.72, 0.67 |
|  |  |  |  |  |  |  |
| **Control statements** | | | | | | |
|  |  |  |  |  |  |  |
| **Statement 4** |  |  |  |  |  |  |
| Synchronous | 1.50 (4.00) | −0.71, 1.78 | −3.00 (5.00) | −2.34, 0.42 | 1.00 (4.00) | −0.93, 1.53 |
| Asynchronous | −2.00 (5.00) | −2.22, −0.41 | −3.00 (4.60) | −2.53, 0.05 | −2.00 (3.30) | −2.64, −0.44 |
|  |  |  |  |  |  |  |
| **Statement 5** |  |  |  |  |  |  |
| Synchronous | −3.00 (3.00) | −2.89, −0.80 | −3.00 (2.00) | −3.00, −1.18 | −3.00 (2.00) | −2.89, 0.72 |
| Asynchronous | −3.00 (4.00) | −2.85, −0.38 | −3.00 (3.50) | −2.68, −0.66 | −3.00 (5.00) | −2.98, −0.82 |
|  |  |  |  |  |  |  |
| **Statement 6** |  |  |  |  |  |  |
| Synchronous | −3.00 (0.00) | −3.31, 1.88 | −3.00 (0.00) | −3.28, −1.52 | −3.00 (0.00) | −3.10, −1.76 |
| Asynchronous | −3.00 (0.00) | −3.24, −1.51 | −3.00 (0.00) | −3.24, −2.08 | −3.00 (0.00) | −3.30, −2.16 |
|  |  |  |  |  |  |  |
| **Statement 7** |  |  |  |  |  |  |
| Synchronous | 1.00 (5.00) | −1.46, 1.26 | −2.00 (4.30) | −2.10, 0.63 | −2.00 (4.00) | −2.02, 0.55 |
| Asynchronous | −2.50 (4.00) | −2.50, 0.14 | −3.00 (3.30) | −2.59, −0.63 | −3.00 (1.50) | −2.87, −1.19 |
|  |  |  |  |  |  |  |
| **Statement 8** |  |  |  |  |  |  |
| Synchronous | −3.00 (0.00) | −3.28, −1.98 | −3.00 (0.00) | −3.22, −2.15 | −3.00 (0.00) | −3.22, −1.72 |
| Asynchronous | −3.00 (0.00) | −3.14, −2.02 | −3.00 (0.00) | −3.19, −2.47 | −3.00 (0.00) | −3.08, −2.58 |
|  |  |  |  |  |  |  |
| **Statement 9** |  |  |  |  |  |  |
| Synchronous | 2.00 (5.00) | −0.30, 2.32 | 0.80 (4.00) | −1.22, 1.11 | 1.00 (5.00) | −1.55, 1.07 |
| Asynchronous | −2.00 (3.80) | −1.90, 0.87 | −2.00 (3.80) | −2.42, −0.34 | 0.00 (3.50) | −1.25, 0.88 |

PH = participant’s own hand

| **Table S3** Median (interquartile range [IQR]) and 95% confidence interval (95% CI) of proprioceptive drift, embodiment-related statements, and control statements in Experiment 2 | | | | | | |
| --- | --- | --- | --- | --- | --- | --- |
|  | **Conditions** | | | | | |
|  | RH-close | | RH-midway | | RH-far | |
| **Drift** | Median (IQR) | 95% CI | Median (IQR) | 95% CI | Median (IQR) | 95% CI |
| Synchronous | 2.00 (2.00) | 1.26, 2.87 | 1.00 (3.50) | 0.19, 2.95 | 0.50 (2.00) | −0.13, 1.66 |
| Asynchronous | 1.00 (2.00) | −0.15, 1.82 | 0.00 (2.50) | −0.12, 1.59 | 1.00 (2.00) | 0.31, 1.49 |
|  | | | | | | |
| **Embodiment-related statements** | | | | | | |
| **Statement 1** |  | |  | |  | |
| Synchronous | 3.00 (0.50) | 2.51, 2.93 | 2.00 (1.50) | 0.98, 2.63 | 2.00 (2.10) | 0.43, 2.56 |
| Asynchronous | −2.00 (1.00) | −2.73, −0.68 | −3.00 (1.00) | −2.98, −1.55 | −3.00 (1.00) | −3.16, 1.72 |
|  |  |  |  |  |  |  |
| **Statement 2** |  |  |  |  |  |  |
| Synchronous | 3.00 (1.00) | 1.81, 3.03 | 2.40 (2.00) | 0.95, 2.63 | 2.00 (4.00) | 0.01, 2.26 |
| Asynchronous | −3.00 (1.00) | −2.90, 1.89 | −3.00 (0.50) | −3.01, −2.46 | −3.00 (0.70) | −3.21, −1.36 |
|  |  |  |  |  |  |  |
| **Statement 3** |  |  |  |  |  |  |
| Synchronous | 3.00 (1.00) | 2.12, 2.90 | 2.00 (1.00) | 0.37, 2.23 | 1.00 (5.00) | −0.74, 1.81 |
| Asynchronous | −1.00 (3.50) | −1.94, 0.41 | −3.00 (1.00) | −2.99, −1.41 | −3.00 (1.00) | −3.07, −2.03 |
|  |  |  |  |  |  |  |
| **Control statements** | | | | | | |
|  |  |  |  |  |  |  |
| **Statement 4** |  |  |  |  |  |  |
| Synchronous | 0.50 (5.00) | −1.06, 1.65 | −2.00 (5.00) | −2.47, 0.25 | −2.80 (4.50) | −2.39, 0.21 |
| Asynchronous | −2.40 (4.00) | −2.44, −0.01 | −2.00 (4.80) | −2.24, 0.48 | −3.00 (4.00) | −2.87, −0.53 |
|  |  |  |  |  |  |  |
| **Statement 5** |  |  |  |  |  |  |
| Synchronous | −3.00 (1.00) | −3.05, −0.84 | −2.90 (4.00) | −2.63, −0.30 | −3.00 (0.00) | −3.12, −1.21 |
| Asynchronous | −3.00 (4.00) | −2.54, 0.23 | −3.00 (1.00) | −3.00, −0.87 | −3.00 (0.00) | −3.21, −2.19 |
|  |  |  |  |  |  |  |
| **Statement 6** |  |  |  |  |  |  |
| Synchronous | −3.00 (0.00) | −3.31, −1.57 | −3.00 (0.00) | −3.27, −1.64 | −3.00 (1.50) | −3.05, −0.98 |
| Asynchronous | −3.00 (0.00) | −3.13, −2.14 | −3.00 (0.00) | −3.39, −1.67 | −3.00 (0.00) | −3.18, −2.01 |
|  |  |  |  |  |  |  |
| **Statement 7** |  |  |  |  |  |  |
| Synchronous | 0.00 (3.50) | −1.33, 0.97 | −3.00 (3.40) | −2.66, −0.35 | −3.00 (2.00) | −2.83, −1.23 |
| Asynchronous | −3.00 (1.00) | −3.03, −1.44 | −3.00 (0.00) | −3.08, −2.58 | −3.00 (0.90) | −3.10, −1.64 |
|  |  |  |  |  |  |  |
| **Statement 8** |  |  |  |  |  |  |
| Synchronous | −3.00 (0.00) | −3.27, −1.13 | −3.00 (1.40) | −2.98, −1.55 | −3.00 (0.00) | −3.27, −2.26 |
| Asynchronous | −3.00 (0.00) | −3.14, −1.86 | −3.00 (1.00) | −3.07, −1.50 | −3.00 (1.00) | −3.12, −1.15 |
|  |  |  |  |  |  |  |
| **Statement 9** |  |  |  |  |  |  |
| Synchronous | 1.60 (1.30) | 1.27, 2.18 | 1.50 (5.00) | −1.35, 1.28 | 1.00 (4.70) | −1.65, 0.81 |
| Asynchronous | 0.00 (5.00) | −1.28, 1.27 | −0.50 (5.00) | −1.63, 0.78 | −2.00 (5.00) | −2.19, 0.33 |

RH = rubber hand

| **Table S4** Median (interquartile range [IQR]) and 95% confidence interval (95% CI) of proprioceptive drift, embodiment-related statements, and control statements in Experiment 3 | | | | | | |
| --- | --- | --- | --- | --- | --- | --- |
|  | **Conditions** | | | | | |
|  | BH-close | | BH-midway | | BH-far | |
| **Drift** | Median (IQR) | 95% CI | Median (IQR) | 95% CI | Median (IQR) | 95% CI |
| Synchronous | 2.00 (2.00) | 1.08, 2.52 | 1.00 (5.00) | 0.50, 3.24 | 1.50 (3.00) | 0.77, 2.56 |
| Asynchronous | 0.00 (2.00) | −1.02, 0.22 | 0.00 (4.00) | −2.65, 0.52 | 0.00 (4.00) | −2.05, 0.72 |
|  | | | | | | |
| **Embodiment-related statements** | | | | | | |
| **Statement 1** |  | |  | |  | |
| Synchronous | 3.00 (2.00) | 1.67, 2.79 | 3.00 (1.60) | 1.64, 2.75 | 2.00 (2.40) | 1.27, 2.40 |
| Asynchronous | −3.00 (1.00) | −2.91, −1.95 | −3.00 (1.00) | −2.90, −1.98 | −3.00 (1.00) | −2.90, −1.63 |
|  |  |  |  |  |  |  |
| **Statement 2** |  |  |  |  |  |  |
| Synchronous | 2.00 (2.00) | 1.64, 2.62 | 2.00 (2.00) | 1.56, 2.63 | 2.00 (2.00) | 1.21, 2.44 |
| Asynchronous | −3.00 (1.00) | −2.98, −1.62 | −3.00 (4.00) | −2.72, −0.74 | −3.00 (1.00) | −2.99, −1.41 |
|  |  |  |  |  |  |  |
| **Statement 3** |  |  |  |  |  |  |
| Synchronous | 2.00 (2.50) | 0.16, 2.31 | 2.00 (2.50) | 0.16, 2.31 | 1.50 (2.50) | 0.10, 2.30 |
| Asynchronous | −1.00 (2.00) | −2.20, −0.33 | −3.00 (2.00) | −2.85, −0.88 | −2.00 (2.00) | −2.27, 0.26 |
|  |  |  |  |  |  |  |
| **Control statements** | | | | | | |
|  |  |  |  |  |  |  |
| **Statement 4** |  |  |  |  |  |  |
| Synchronous | −3.00 (4.00) | −2.33, 0.13 | −3.00 (3.00) | −2.70, −0.43 | −1.00 (3.50) | −2.26, 0.06 |
| Asynchronous | −3.00 (2.00) | −2.83, −0.70 | −2.50 (1.00) | −2.78, −0.89 | −3.00 (4.00) | −2.55, −0.31 |
|  |  |  |  |  |  |  |
| **Statement 5** |  |  |  |  |  |  |
| Synchronous | −3.00 (1.00) | −3.07, −1.20 | −3.00 (0.00) | −3.18, −1.62 | −3.00 (1.00) | −2.99, −1.03 |
| Asynchronous | −3.00 (3.00) | −2.74, −0.86 | −3.00 (1.00) | −3.11, −1.56 | −3.00 (1.00) | −3.02, −1.25 |
|  |  |  |  |  |  |  |
| **Statement 6** |  |  |  |  |  |  |
| Synchronous | −3.00 (0.00) | −3.17, −1.37 | −3.00 (0.00) | −3.32, −1.88 | −3.00 (1.00) | −3.09, −1.84 |
| Asynchronous | −3.00 (1.00) | −3.07, −1.39 | −3.00 (2.00) | −3.04, −1.22 | −3.00 (2.00) | −2.94, −1.33 |
|  |  |  |  |  |  |  |
| **Statement 7** |  |  |  |  |  |  |
| Synchronous | −2.00 (3.00) | −2.58, −0.42 | −2.00 (3.50) | −2.71, −0.42 | −2.00 (5.00) | −2.16, 0.69 |
| Asynchronous | −3.00 (1.00) | −3.04, −1.10 | −3.00 (1.00) | −3.11, −0.69 | −3.00 (2.00) | −2.88, −0.80 |
|  |  |  |  |  |  |  |
| **Statement 8** |  |  |  |  |  |  |
| Synchronous | −3.00 (0.00) | −3.16, −1.91 | −3.00 (1.00) | −3.12, −1.95 | −3.00 (2.00) | −3.01, −1.60 |
| Asynchronous | −3.00 (0.00) | −3.12, −1.68 | −3.00 (1.00) | −3.06, −2.14 | −3.00 (2.00) | −2.90, −1.50 |
|  |  |  |  |  |  |  |
| **Statement 9** |  |  |  |  |  |  |
| Synchronous | 1.00 (4.00) | −0.62, 1.82 | 2.00 (5.00) | −1.03, 1.70 | 1.00 (3.50) | −0.66, 1.79 |
| Asynchronous | 0.50 (5.00) | −1.47, 1.18 | −1.00 (5.00) | −1.87, 0.80 | −1.00 (5.00) | −1.68, 0.87 |

BH = both hands

**References**

Abdulkarim, Z., & Ehrsson, H. H. (2016). No causal link between changes in hand position sense and feeling of limb ownership in the rubber hand illusion. *Attention, Perception, & Psychophysics, 78*(2), 707–720. doi:10.3758/s13414-015-1016-0
